# A powerful score-based statistical test for group difference in weighted biological networks

**DOI:** 10.1186/s12859-016-0916-x

**Published:** 2016-02-12

**Authors:** Jiadong Ji, Zhongshang Yuan, Xiaoshuai Zhang, Fuzhong Xue

**Affiliations:** Department of Biostatistics, School of Public Health, Shandong University, PO Box 100, Jinan, 250012 Shandong China

**Keywords:** Network medicine, Systems epidemiology, Score-based statistical test, Network comparison

## Abstract

**Background:**

Complex disease is largely determined by a number of biomolecules interwoven into networks, rather than a single biomolecule. A key but inadequately addressed issue is how to test possible differences of the networks between two groups. Group-level comparison of network properties may shed light on underlying disease mechanisms and benefit the design of drug targets for complex diseases. We therefore proposed a powerful score-based statistic to detect group difference in weighted networks, which simultaneously capture the vertex changes and edge changes.

**Results:**

Simulation studies indicated that the proposed network difference measure (*NetDifM*) was stable and outperformed other methods existed, under various sample sizes and network topology structure. One application to real data about GWAS of leprosy successfully identified the specific gene interaction network contributing to leprosy. For additional gene expression data of ovarian cancer, two candidate subnetworks, *PI3K-AKT* and *Notch* signaling pathways, were considered and identified respectively.

**Conclusions:**

The proposed method, accounting for the vertex changes and edge changes simultaneously, is valid and powerful to capture the group difference of biological networks.

**Electronic supplementary material:**

The online version of this article (doi:10.1186/s12859-016-0916-x) contains supplementary material, which is available to authorized users.

## Background

From the perspective of network medicine, a disease phenotype is rarely a consequence of an abnormality in a single biomolecule (e.g. RNA, protein, metabolite), but reflects various pathobiological processes that interact in a complex network [1]. One single factor can exert certain effects on disease when studying it alone, while this effect may be vanished when studying it within one network or pathway [2], and vice versa. Therefore, biomolecules should be studied in the context of biological systems they are involved in [3]. Perhaps the abstraction for a biological system is network, such as transcriptional regulatory networks, signal transduction networks, protein interaction networks and metabolic networks [4]. In the biological networks, the vertices represent biomolecules, and the edges represent functional, causal or physical interactions between the vertices. Different types of networks are often used to represent diverse types of biological processes, each of which stores information about levels and interactions related to specific biomolecules [5]. In fact, different physiological conditions may manifest as different networks. Moreover, complex disease are multi-factorial and analyzing the individual components is insufficient, so it is essential to dissect how these components interact with each other and weave into one network, and how these interactions differ with respect to disease status. Statistical comparison of group difference in biological networks or pathways can provide new insight into the underlying disease mechanism, and have extensive biomedical and clinical applications [6–10]. For instance, a better understanding of the effects of molecular interconnectedness on disease progression may lead to superior identification of disease related biomolecules and pathways, which may further offer more effective targets for drug development in a cost-effective and timely manner.

On the other hand, identifying biological and environmental causes of human diseases has always been one of the central concerns in epidemiology. However, traditional epidemiology has been pejoratively labeled as the “black box” epidemiology [11], and increasingly suffered from criticism partly due to the fact that too much attention has been paid to the identification of a single risk factor rather than the network or pathway related to a disease, which led to difficulty to deeply explore disease mechanism [12]. It is highly desirable to unlock the black box underlying observed associations and to illuminate the biological interaction mechanisms of disease-related components hiding behind the black box. There are unmet needs to access multi-level omics data on the population level. Thanks to the development of recent technological advances in high-throughput omics platforms, we can enable the acquisition of omics data at unprecedented speed and amounts, and further integrate various omics data with traditional epidemiology to promote the development of systems epidemiology [12, 13]. It offers the potential to provide new insight into the underlying disease mechanisms in breadth and depth at human population level. Under the framework of systems epidemiology, the focus has been shifted from identification of single factor to exploration of specific networks or pathways contributing to disease [14, 15].

In a word, it is in great needs to do statistical comparison of biological networks. So far, several methods have been proposed to utilize network topology information to carry out various biomedical tasks. Langfelder et al. [16] provided several measures for comparing network topologies for weighted correlation networks. Zhang et al. [17] proposed a differential dependency network analysis to detect topological changes in transcriptional networks between subclasses of breast cancer. Valcarcel et al. [18] introduced a formal statistical method for the differential analysis of molecular pair-wise associations via network representation. Recently, Yates et al. [19] developed an additive element-wise-based dissimilarity measure for biological network hypothesis tests. However, most of above methods mainly focus on the difference of network topology and are unable to account for the changes of vertices. Although in most situations, the differences of single vertices-wise or edges-wise may be weak, their aggregated differences can be quite strong. It will undoubtedly lose statistical power to only consider the connection with the topological difference between two networks. Meanwhile, non-parametric permutation procedures are commonly employed to perform analysis in most existed methods, which were inevitably time-consuming, especially for big data.

The premise for networks or pathways comparison is to make clear the cause of biological network difference. Generally, both changes in the nodes level (e.g. the magnitude of each gene’s expression change in regulation network), and changes in the edges (e.g. the strength of connection) can lead to the whole network difference. Reverter et al. [20] presented an analytical procedure to simultaneously identify genes that were differentially expressed (DE) as well as genes that are differentially connected (DC) for unweighted networks. Their methods depend heavily on the specific definition of DE and DC, and the two-component mixture of bi-variate normal distribution may be violated in other biological networks, though it may be reasonable in gene expression network. Furthermore, weighted (correlation-based) networks are commonly encountered and increasingly relevant in biological applications [16, 21–23]. Statistical methods for detecting the group difference in weighted biological networks are still in great demand.

In this article, we proposed a new score-based network difference measure (*NetDifM*) as a powerful test statistic to detect group difference in weighted networks, which simultaneously capture the difference of vertices and edges. Various simulations were conducted to evaluate its type I error and statistical power, compared with other existed method. Two real data sets about GWAS of leprosy and gene expression of ovarian cancer were further analyzed to show their performance in practice.

## Methods

### Statistical model

A weighted biological network can be modeled as an undirected graph *G* = (*V*, *E*), where *V* is the set of vertices (sometimes referred to nodes) and *E* is the set of edges (also called connections). Two vertices, representing biomolecules, are connected by an undirected edge if there is an association between them. Each edge can be assigned a weight resembling the strength of evidence for the association.

We denote the two networks in two groups (cases and controls) by *G*^*D*^ and *G*^*C*^ respectively, suppose both *G*^*D*^ and *G*^*C*^ have the same number of vertices (*M*) and edges (*K*), the null hypothesis test is *H*_0_ : *G*^*D*^ = *G*^*C*^. Let *V*(*G*^*D*^) and *E*(*G*^*D*^) denote the set of all vertices and edges in *G*^*D*^, *x*_*i*_^*D*^*x*_*j*_^*D*^ indicate the edge ${x}_i^D-{x}_j^D$ (*i* ≠ *j*, *i*, *j* = 1, 2, ⋯, *M*), *β*_*ij*_^*D*^ represent the strength of association between *x*_*i*_^*D*^ and *x*_*j*_^*D*^ if *x*_*i*_^*D*^*x*_*j*_^*D*^ existed. For individual *l* (*l* = 1, 2, ⋯, *N*), the trait value is denoted as *Y*_*l*_, $$ {Y}_l=\left\{\begin{array}{l}1,\kern0.4em l\in case\\ {}0\kern0.1em ,\kern0.2em l\in control\end{array}\right. $$ and the *i*^th^ vertex is denoted as *x*_*li*_. Under *H*_0_, networks in two groups are identical not only in the average vertices levels but also in the connection strength. The score test vector of vertices is *D*^*V*^ = (*D*_1_^*V*^, *D*_2_^*V*^, ⋯, *D*_*M*_^*V*^)^*T*^, where ${D}_i^V={\displaystyle {\sum}_{l=1}^N\left({Y}_l-\overline{Y}\right){x}_{li}}$ measures the contribution of vertex *x*_*i*_ to the disease. Analogously, the score test vector of edges is *D*^*E*^ = (*D*_1_^*E*^, *D*_2_^*E*^, ⋯, *D*_*K*_^*E*^)^*T*^, where ${D}_k^E={\displaystyle {\sum}_{l=1}^N\left({Y}_l-\overline{Y}\right)\left({x}_{li}-{\overline{x}}_i\right)\left({x}_{lj}-{\overline{x}}_j\right)}$ measures the contribution of connection strength between *x*_*i*_ and *x*_*j*_ (i.e. the *k*^th^ edge) to the disease. Then the proposed overall network difference measure can be defined as

$$ NetDifM={D}^T{\varSigma}^{-1}D, $$

where $$ D=\left(\begin{array}{l}{D}^V\\ {}{D}^E\end{array}\right), $$*Σ* = cov(*D*) = (*σ*_*pq*_)_(*M* + *K*) × (*M* + *K*)_, *p*, *q* = 1, 2, ⋯, (*M* + *K*). The estimated covariance matrix of *D* can be represented as $$ \left(\kern-.9em \begin{array}{cc}{\varSigma}_V& {\varSigma}_{VE}\\ {}\kern1em {\varSigma}_{VE}^T\kern1em & {\varSigma}_E\end{array}\right) $$ and calculated as follows,

1) For *Σ*_*V*_, *p*, *q* = 1, 2, ⋯, *M*,

$$ {\sigma}_{pq}={\displaystyle {\sum}_{l=1}^N{\left({Y}_l-\overline{Y}\right)}^2\operatorname{cov}\left({X}_p,{X}_q\right)},{X}_p=\left({x}_{1p},{x}_{2p},\cdots, {x}_{Np}\right); $$

2) For *Σ*_*E*_, *p*, *q* = *M* + 1, *M* + 2, ⋯, *M* + *K*,

$$ {\sigma}_{pq}={\displaystyle {\sum}_{l=1}^N{\left({Y}_l-\overline{Y}\right)}^2\operatorname{cov}\left({Z}_p,{Z}_q\right)},{Z}_p=\left({X}_i-{\overline{X}}_i\right)\times \left({X}_j-{\overline{X}}_j\right); $$

3) For *Σ*_*VE *_, *p* = 1, 2, ⋯, *M *, *q* = *M* + 1, *M* + 2, ⋯, *M* + *K*

$$ {\sigma}_{pq}={\displaystyle {\sum}_{l=1}^N{\left({Y}_l-\overline{Y}\right)}^2\operatorname{cov}\left({X}_p,{Z}_q\right)}. $$

Naturally, for a large sample size, *NetDifM* has a centered *χ*^2^(*M* + *K*) distribution under the null hypothesis (The derivation of *NetDifM* see Additional file 1). When sample size is small, a permutation procedure can be conducted as follows to get the empirical *P* value and assess the statistical significance. (1) calculate the test statistic *NetDifM* from the original sample; (2) randomly assign subjects to one of two groups, the sample size for each group keeps the same as the original data; (3) perform the above steps *Q* times and calculate the test statistic for each repeated sample, *NetDifM*_i_^*^, *i* = 1, 2, ⋯, *Q*; (4) estimate the *P* value according to $p- value=\frac{1}{Q}{\displaystyle \sum_{i=1}^QI\left( NetDif{M}_i^{*}> NetDifM\right)}$, where *I*(•) is the indicator function.

Intuitively, considering the elements of one network are not more than vertices and edges, an element-wise measure may be expected to have the ability to identify the group difference in biological networks. A vertices and edges wise difference measure (*VEWDM*), through the simple summation of vertices difference and edges, can be constructed as

$$ VEWDM=\frac{1}{M}{\displaystyle \sum_{i=1}^M{T}_i^2+\frac{1}{K}{\displaystyle \sum_{i=1}^M{\displaystyle \sum_{j\ne i}^M{U}_{ij}^2}}} $$

where ${T}_i=\frac{{\overline{x}}_i^D-{\overline{x}}_i^C}{\sqrt{\operatorname{var}\left({\overline{x}}_i^D\right)+\operatorname{var}\left({\overline{x}}_i^C\right)}}$, ${\overline{x}}_i^D$ and ${\overline{x}}_i^C$ indicate the sample mean of *x*_*i*_ in *G*^*D*^ and *G*^*C*^ respectively; ${U}_{ij}=\frac{\beta_{ij}^D-{\beta}_{ij}^C}{\sqrt{\operatorname{var}\left({\beta}_{ij}^D\right)+\operatorname{var}\left({\beta}_{ij}^C\right)}}$, when the strength of edges are quantified by the Pearson correlations *r*_*ij*_, ${U}_{ij}=\left({z}_{ij}^D-{z}_{ij}^C\right)/\sqrt{\frac{1}{n_D-3}+\frac{1}{n_C-3}}$, *z*_*ij*_ are the Fisher-transforms of the correlations ${z}_{ij}=\frac{1}{2} \ln \frac{1+{r}_{ij}}{1-{r}_{ij}}$, *n*_*D*_ and *n*_*C*_ are the corresponding sample sizes. The proposed *VEWDM* seems to be the linear combination of some chi-square statistics. Actually, the asymptotic theoretical properties have been explored for the linear combination of independent chi-squares [24]. Nevertheless, it is quite complex and difficult here to obtain the asymptotic distribution of *VEWDM*, since the correlations between different vertices and different edges statistics (*T*_*i*_ and *U*_*ij*_) highly depend on the specific network structure. In other words, the asymptotic properties might be network-specific. To solve this problem, we adopted the strategy of a permutation test to make statistical inference.

### Simulation

Simulations were designed to evaluate the type I error rate and statistical power, and to compare the performance of *NetDifM*, *VEWDM* and *Yates’D* (recently proposed dissimilarity measure in Yates et al. [19]) under different sample size and network topological structure. The statistical power is defined as the probability that the test correctly rejects the null hypothesis (*H*_0_) when the alternative hypothesis (*H*_1_) is true. It can be estimated from the empirical distribution as the proportion of observations for which the *p*-value is less than given nominal level (*α* = 0.05). For the specific network with *M* vertices and *K* edges, the simulated *M*-dimensional variables (vertices) were generated from a multivariate normal distribution *N*_*M*_(**μ**, **Σ**) with mean vector **μ** and covariance matrix **Σ** using the R “*mvtnorm*” package. We specified the mean vector **μ** = (*μ*_1_, *μ*_2_, ⋯, *μ*_*M*_) and covariance matrix **Σ** = (*I*_*ij*_*β*_*ij*_)_*M* × *M*_, where $$ {I}_{ij}=\left\{\begin{array}{l}1,\kern0.4em {x}_i{x}_j\in E(G)\\ {}0,\kern0.3em {x}_i{x}_j\notin E(G)\kern0.1em \end{array}\right. $$, *i* ≠ *j*, *i*, *j* = 1, 2, ⋯, *M* was the indicator function.

**Fig. 1:**
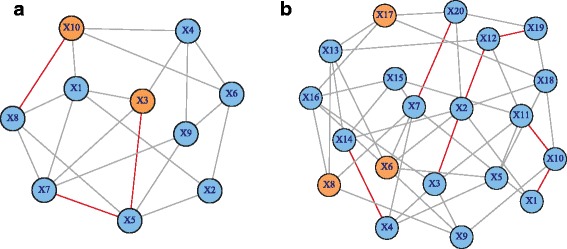
Weighted biological networks. **a** An assumed network including ten vertices and 21 edges. **b** An assumed network including 20 vertices and 45 edges

Under the null hypotheses (*H*_0_), the data was generated by setting **μ**^*D*^ = **μ**^*C*^ and *I*_*ij*_^*D*^*β*_*ij*_^*D*^ = *I*_*ij*_^*C*^*β*_*ij*_^*C*^. 1000 simulations were repeated to assess the type I error of the above methods given various sample sizes under different network scale, including network with ten vertices and 21 edges (Fig. 1a) and another one with 20 vertices and 45 edges (Fig. 1b). Under the alternative hypotheses, three scenarios were considered.

For scenario 1, only vertices (average levels) were different between *G*^*D*^ and *G*^*C*^. We simulated two vertices difference with *μ*_3_^*D*^ − *μ*_3_^*C*^ = 0.2 and *μ*_10_^*D*^ − *μ*_10_^*C*^ = 0.2 under the network topological structure as in Fig. 1a. Three vertices difference with *μ*_6_^*D*^ − *μ*_6_^*C*^ = 0.1, *μ*_8_^*D*^ − *μ*_8_^*C*^ = 0.2 and *μ*_17_^*D*^ − *μ*_17_^*C*^ = 0.2 were also designed under the relative larger scale network as in Fig. 1b.

For scenario 2, only edges (connection strength) were different between *G*^*D*^ and *G*^*C*^. We simulated three edges difference with *β*_35_^*D*^ − *β*_35_^*C*^ = − 0.2, *β*_57_^*D*^ − *β*_57_^*C*^ = 0.2 and *β*_8,10_^*D*^ − *β*_8,10_^*C*^ = 0.2 under the network topological structure as in Fig. 1a. Seven edges difference with *β*_10,11_^*C*^ − *β*_10,11_^*D*^ = − 0.2, *β*_1,10_^*C*^ − *β*_1,10_^*D*^ = *β*_2,12_^*C*^ − *β*_2,12_^*D*^ = *β*_4,14_^*C*^ − *β*_4,14_^*D*^ = *β*_12,19_^*C*^ − *β*_12,19_^*D*^ = 0.2 and *β*_23_^*C*^ − *β*_23_^*D*^ = *β*_7,20_^*C*^ − *β*_7,20_^*D*^ = 0.1 were also designed under the relative larger scale network as in Fig. 1b.

For scenario 3, both vertices and edges were designed to be different between *G*^*D*^ and *G*^*C*^. Under the topology structure as in Fig. 1a, we combined the settings in scenario 1 and scenario 2 (the difference only existed for orange vertices and red edges), so as for the topology structure as in Fig. 1b.

For each scenario, 1000 replicates were used to evaluate statistical power. *P*-values of the proposed *NetDifM* were assessed using both the asymptotic distribution and the empirical null distribution obtained from 1000 times permutations.

It is necessary to assess the performance of the proposed statistics, given the deviation from the normal distribution. For the network with ten vertices and 21 edges, we designed the following two scenarios, (i) conduct the exponential transformation for five vertices randomly chosen among the ten vertices; (ii) do the exponential transformation for all ten vertices. For each scenario, we evaluate the type I error rate and statistical power under the same three scenarios mentioned as above.

Furthermore, we also provided estimated computing time under different network with sample size 200 and 1000 permutations, using one laptop as an Intel PentiumT4400 with a 2.2 GHz CPU and 2 GB RAM.

### Application

#### GWAS data of leprosy

**Fig. 2:**
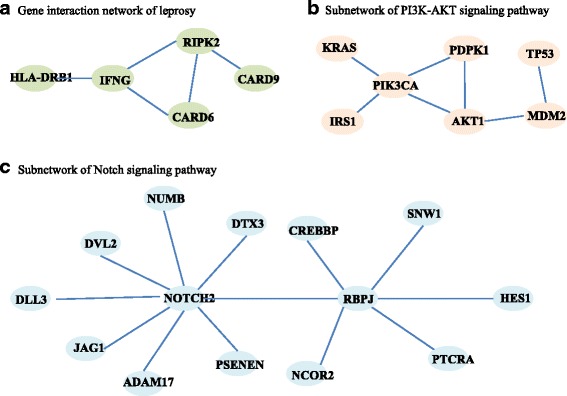
Gene networks. **a** Gene interaction network of leprosy. **b** Subnetwork of *PI3K-AKT*signaling pathway. **c** Subnetwork of *Notch* signaling pathway

By Ingenuity Pathways Analysis, a plausible biologic network underlying susceptibility to leprosy was provided for depicting the functional relationship between some susceptibility genes identified from GWAS of leprosy [25]. Using the initial GWAS data with 706 cases and 514 controls, we attempted to detect the difference of the networks including genes *CARD6*, *HLA-DRB1*, *RIPK2*, *CARD9* and *IFNG*. All participants provided written informed consent, and the study was approved by the ethics committees of Shandong Academy of Medical Science [25]. These five genes located on different chromosomes and totally contained 914 SNPs (see in Additional file 1: Table S1), with network structure given in Fig. 2a. Since each gene contained several SNPs, we first employed principal component analysis and conducted the statistical network comparison by treating the first principal component as the network vertices.

#### Gene expression data of ovarian cancer

Tothillet al. [26] used high-density expression oligonucleotide microarrays for profiling 285 well-annotated serous and endometrioid invasive ovarian, fallopian tube, and peritoneal cancers. The subjects were divided into a C1 subtype, with 83 patients, and a C2–C6 subtype, with 168 patients. Complete expression data are available on GEO (accession GSE9899). The proposed method was also applied to detect the network difference between these two groups (C1 versus C2–C6). Here we studied two specific pathways (*PI3K-AKT* signaling pathway and *Notch* signaling pathway) reported in the literatures [27–29] to be relevant to ovarian cancer. The subnetwork of *PI3K-AKT* signaling pathway from the KEGG pathway database with 7 genes contained 26 probe sets (see in Additional file 1: Table S2) was abstracted into network with topological structure shown in Fig. 2b. The subnetwork of *Notch* signaling pathway with 14 genes contained 45 probe sets (see in Additional file 1: Table S2) was abstracted into network with topological structure shown in Fig. 2c. All probe sets corresponding to the same gene symbol were first averaged to obtain gene-level expression measurements.

## Results

### Simulation

Shown in Table 1 are the estimated type I error rates of the proposed *NetDifM*, *VEWDM*, *NetDifM* based on permutation (*NetDifM*pm) and *Yates’D* under different sample sizes. It reveals that all type I error rates based on permutation procedure are close to given nominal level (*α* = 0.05). *NetDifM* tended to be slightly conservative under small sample size, while using the asymptotic distribution maintains a good control of type I error rate under large sample size.

**Table 1:** Type I error rates of four methods

Sample size	*NetDifM*	*NetDifM*pm	*VEWDM*	*Yates’D*
10 vertices & 21 edges				
100	0.014	0.052	0.050	0.047
200	0.031	0.053	0.051	0.048
300	0.044	0.058	0.057	0.043
500	0.043	0.047	0.046	0.050
800	0.055	0.058	0.044	0.059
20 vertices & 45 edges				
200	0.025	0.048	0.048	0.045
300	0.034	0.051	0.051	0.054
500	0.041	0.055	0.055	0.056
800	0.045	0.056	0.051	0.054
1000	0.052	0.055	0.051	0.047

**Fig. 3:**
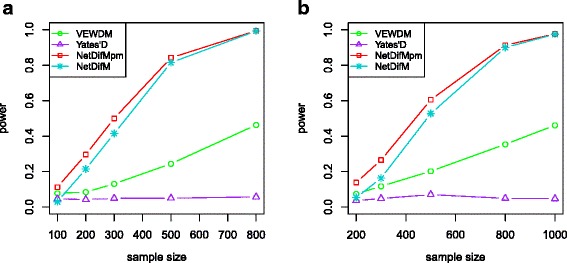
The statistical power of the four methods under the scenario only vertices was different between two groups. **a** The power under the network topological structure as in Fig. 1a. **b** The power under the network topological structure as in Fig. 1b

Figure 3a indicates the statistical power under scenario 1 when only vertices changed with the network topological structure demonstrates in Fig. 1a. As expected, *Yates’D* has no power due to that it can only capture the edge change. *NetDifM* is substantially more powerful than *VEWDM*, and it is slightly less powerful than its permutation-based type. Similar trend could also be found under the relative larger scale network (Fig. 3b).

**Fig. 4:**
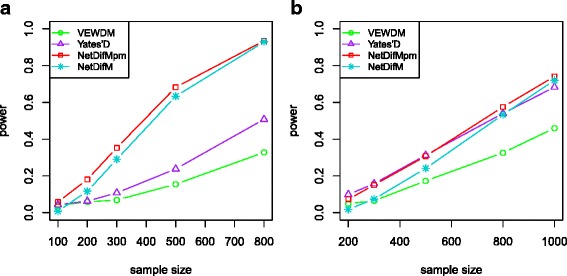
The statistical power of the four methods under the scenario only edges was different between two groups. **a** The power under the network topological structure as in Fig. 1a. **b** The power under the network topological structure as Fig. 1b

Shown in Fig. 4 is the performance under scenario 2 (only edges change). The statistical power of all methods monotonically increases with sample size. *NetDifM* has much higher power than that of *VEWDM* and *Yates’D*. The power of *NetDifM* and *Yates’D* keep almost the same in the larger scale network (Fig. 4b).

**Fig. 5:**
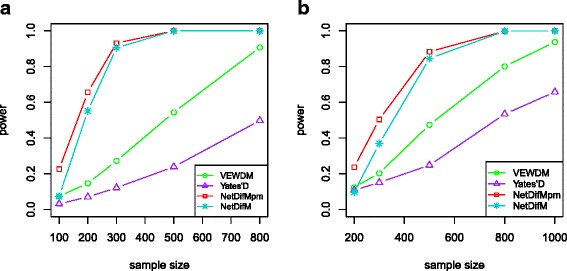
The statistical power of the four methods under the scenario both vertices and edges were different between two groups. **a** The power under the network topological structure as in Fig. 1a. **b** The power under the network topological structure as in Fig. 1b

Figure 5 illustrates the statistical power under the scenario 3 (both edges and vertices change). Both *NetDifM* and *VEWDM* are much more powerful than *Yates’D*, and *NetDifM* still has the best performance.

To evaluate the scalability and computational efficiency of the proposed methods, we also conducted simulations using a larger network with 40 vertices and 54 edges (see in Additional file 1: Figure S1). It is clear that the proposed *NetDifM* still have the best performance (see in Additional file 1: Figure S2; Additional file 1: Table S3).

**Fig. 6:**
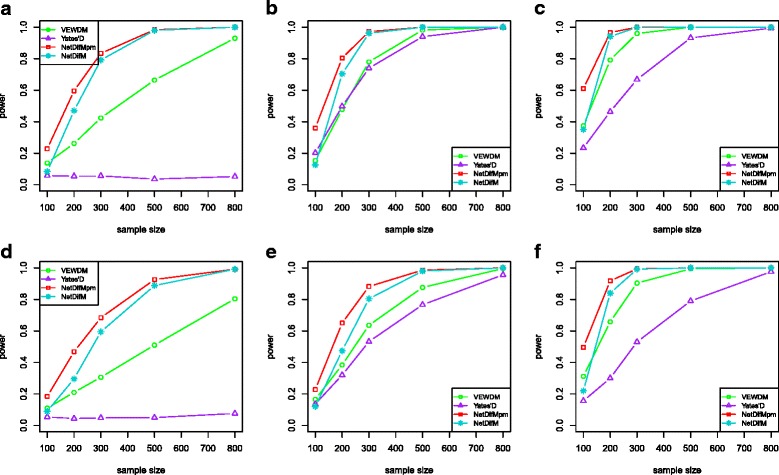
The statistical power of the four methods given the deviation from the normal distribution. **a**, **b**, **c** The power under the scenario conducting the exponential transformation for five vertices, when only vertices change (**a**), only edge changes (**b**), both vertices and edges change (**c**). **d**, **e**, **f** The power under the scenario doing the exponential transformation for all ten vertices, when only vertices change (**d**), only edge changes (**e**), both vertices and edges change (**f**)

Figure 6 indicates the results given the deviation from the normal distribution, where the proposed statistics still hold the relative better performance than other method.

Table 2 presents the estimated computing time. It indicates that the proposed *NetDifM* indeed runs fast, and the computational time increases as the network become larger.

**Table 2:** Computing time (seconds) of four methods with sample size 200 and 1000 permutations

	*NetDifM*	*NetDifM*pm	*VEWDM*	*Yates’D*
Network1	0.0015	0.91	0.76	3.83
Network2	0.0034	2.04	1.07	6.95
Network3	0.0064	3.92	2.16	12.37

Network1 ten vertices and 21 edges; Network2 20 vertices and 45 edges;

Network3 40 vertices and 54 edges

### The results of application

Network difference analysis for both the GWAS of leprosy and gene expression data of ovarian cancer further confirm in practice that the proposed *NetDifM* captured the network changes. Shown in Table 3 are the results of the proposed *NetDifM* and other methods for detecting the network difference between two groups. The difference of gene interaction network with 5 genes can be detected significantly at *α* = 0.05 by *NetDifM*, *NetDifM*pm and *VEWDM.*

**Table 3:** *P*-values of the four methods for the two real data sets (1000 times permutation)

Networks	*NetDifM*	*NetDifM*pm	*VEWDM*	*Yates’D*
Leprosy	0.003	0.008	0.006	0.230
Ovarian (*PI3K-AKT*)	0.006	0.008	0.017	0.465
Ovarian (*Notch*)	2.89 × 10^– 6^	<0.001	<0.001	0.031

Group difference of the subnetwork of *PI3K-AKT* signaling pathway was detected significantly at *α* = 0.05 by *NetDifM*, *NetDifM*pm and *VEWDM*. When applied to the subnetwork of *Notch* signaling pathway, all four methods can detect the network difference significantly (Table 3). Shown in Table 4 are the results of the proposed *NetDifM* and other methods for detecting the specific vertices, treating a vertex as well as its connected edge as a network, under1000 permutation times.

**Table 4:** *P*-values of the four methods for detecting the genes in the *PI3K-AKT* signaling pathway and *Notch* signaling pathway

Gene	*NetDifM*	*NetDifM*pm	*VEWDM*	*Yates’D*
*PI3K-AKT* signaling pathway				
*KRAS*	0.19521	0.190	0.551	0.499
*PIK3CA*	0.49654	0.531	0.531	0.891
*IRS1*	0.06622	0.066	0.031	0.207
*PDPK1*	0.05108	0.042	0.032	0.143
*AKT1*	0.13639	0.146	0.065	0.712
*MDM2*	0.03568	0.035	0.011	0.484
*TP53*	0.08385	0.083	0.136	0.102
*Notch* signaling pathway				
*DLL3*	0.05909	0.062	0.081	0.559
*DTX2*	0.42435	0.412	0.202	0.122
*CREBBP*	0.20470	0.220	0.191	0.650
*PTCRA*	0.51705	0.507	0.421	0.192
*JAG1*	0.03144	0.027	0.036	0.142
*DVL2*	0.00124	<0.001	<0.001	0.639
*SNW1*	0.71303	0.706	0.309	0.124
*HES1*	0.01032	0.002	0.035	0.995
*RBPJ*	0.00001	<0.001	<0.001	0.646
*NOTCH2*	0.00014	<0.001	<0.001	0.095
*PSENEN*	0.42405	0.435	0.102	0.032
*ADAM17*	0.00701	0.009	0.009	0.326
*NUMB*	0.10758	0.098	0.065	0.100
*NCOR2*	0.00021	<0.001	<0.001	0.344

## Discussion and conclusions

Complex disease is largely determined by a number of biomolecules interwoven into networks, rather than a single biomolecule. Group-level comparison of network properties (vertices level and the strength of connection between vertices) may shed light on underlying biological processes or disease mechanisms, and benefit the design of drug targets and drug combination for the therapy of complex diseases. Meanwhile, although the conventional single-based paradigm has successfully identified a list of risk factors, one common sense is that there still exist an intermediate “black box” between the exposures and the disease phenotypes (end point observations). In the “black box”, various risk factors weaved into complicated biological networks dominating the disease occurrence, development and prognosis. Recent advances in high-throughput technologies and omics resources are revolutionizing biomedical research, and allow a transition from the traditional paradigm for biological and epidemiological studies of complex diseases to a new paradigm based on systems epidemiology [12, 13, 15]. Under this framework, network-based analysis has been integrated into observational study designs to organize the interdependencies of biomolecules and other factors at a human population level, expecting to open the “black box”. A key but inadequately addressed issue is still to develop valid statistical method to test possible differences of the networks between two groups.

In our previous study [15], we have developed a statistical method for detecting the pathway effect contributing to disease, mainly under the framework of systems epidemiology. However, this method is limited to the pathway with chain structure, and can only capture the edge changes while omitting the vertex changes. Meanwhile, the nonparametric bootstrap method has to be used to obtain the significance. At present study, we proposed a score-based powerful statistical test to detect the significant changes in biological networks between two different conditions (e.g. health and disease). It can simultaneously capture the vertex changes and edge changes. Various simulations were conducted to assess the reliability and statistical power of the proposed method. It indicated that both *NetDifM* and *VEWDM* were much more powerful than *Yates’D*, and *NetDifM* kept the best performance under various scenarios (Figs. 3 and 6), and it can indeed capture the perturbation of vertices and edges in the network simultaneously. One strength for *NetDifM* is that we can obtain its theoretic property, and thus can avoid the high computation burden. As expected, the proposed *NetDifM* indeed runs fast (Table 2). The *VEWDM* was used Fisher r-to-z transformation to identify significant differences between two correlations. Fukushima et al. [30] also developed an R package to identify differential correlations between two conditions based on Fisher’s z-test which affords users a simple and effective framework in omics data. The *VEWDM* can be treated as a global measure to detect the group difference of networks between two conditions, accounting for not only edges difference but also vertices difference. Even though one is interested in testing particular vertex or edge rather than the whole network, its connected edge should also be considered.

Two real data sets analyses further highlighted that *NetDifM* had more advantage in practice. In the GWAS data of leprosy, we detected a candidate gene interaction network containing five genes. For the gene expression data of ovarian cancer, two candidate subnetworks, *PI3K-AKT* signaling pathway and *Notch* signaling pathway, respectively were considered and identified, suggesting that the proposed method is capable of identifying differential gene expression and gene-gene co-expression patterns, which are certainly helpful for us to further understand the underlying disease mechanism. Rao et al. [31] reported that combined overexpression of *OVA66* and *MDM2* promotes oncogenesis by enhancing activation of the IGF-1R–ERK1/2 signaling pathway, and *JAG1* enhances ovarian cancer cell growth and cisplatin-resistance [32]. The expression of *HES1* is confirmed to be strongly associated with the pathogenesis of ovarian endometriomas [33]. Meanwhile, decreased *NOTCH2* expression is associated with the poorly differentiated serous epithelial ovarian carcinoma histology [34]. *RBPJ* underexpression in ovarian tumor tissue relative to matched normal tissue [35]. Moreover, *ADAM17* is one of the several metalloproteinases that play a key role in epidermal growth factor receptor signalling and can be a potential target antigen to devise novel immunotherapeutic strategies against ovarian cancer [36]. The *PI3K-AKT* and *Notch* pathways and their abundant associated genes comprise complicated networks, which play a significant role in the progressive growth of tumor cells.

Network difference can result from not only changes of vertices but also changes of edges, and the changes of vertices-wise and edges-wise are often closely related. For instance, differential expression of genes may be due to either mutation of its own gene or the effects of expression changes of other genes in the network. However, the degree of differential expression of one gene due to its own mutation is often lower than affected by expressions of upstream genes in the network [37]. Reverter et al. [20] presented an analytical procedure to simultaneously identify differential gene expression and connectivity for unweighted gene network. In their work, an edge between two genes is established if the absolute value of the correlation coefficient exceeds a fixed threshold. Consequently, if we set the threshold less than 0.5, and the correlation coefficient between gene A and gene B is 0.9 in cases and 0.5 in controls, then the connection between gene A and gene B is treated as no difference between cases and controls. While in this situation, there exists a difference of the strength of connection between gene A and gene B among cases and controls, given our methods focus mainly on weighted biological networks.

Furthermore, the covariance structure between vertex changes and edge changes has been embedded into the proposed score-based network difference measure. In addition, one would be more interested in testing particular vertex or edge (genes or metabolites) rather than the whole network or pathway. Actually, a vertex as well as its connected edge can be treated as a subnetwork, and the proposed network difference method can easily be extended to identify the specific vertices. Even though some local interventions were often generated to prevent and cure a particular disease, it is essential to understand the global system. The ‘think globally, act locally’ paradigm should be strongly embedded into our mind [1].

One limitation in our paper is that we assume the network topological structure is fixed, and little attention has been paid on the network structure learning problem. Constructing network structure means determining every possible edge with highest degree of data matching, and often one joint probability distribution of a number of variables can reflect more than one network structure. Usually, combining experimental evidence with their experience, most biologists and clinical researchers have a growing awareness of the interplay between the biological components and can depict more or less the specific network or pathway for the corresponding biological process. Meanwhile, numerous databases (e.g. KEGG, GO, I2D) can be further borrowed to establish the network structure. The proposed *NetDifM* will do not work in its current version under the scenario when the covariance matrix is not invertible. One possible solution is to first apply a shrinkage strategy to simplify the network, and then adopt the proposed statistic. For instance, we can first remove those edges if the correlation between the two linked vertices is smaller than a predefined threshold, and then apply the proposed test to the remaining network.

Statistical comparisons of group difference in biological networks are highly desirable. The proposed network difference measure was valid and powerful to detect biological network difference. R code to perform *NetDifM*, *NetDifM*pm and *VEWDM* is provided in the Additional file 2. 

### Availability of supporting data

The gene expression data of ovarian cancer were downloaded from the GEO datasets (http://www.ncbi.nlm.nih.gov/geo/query/acc.cgi?acc=GSE9899).

## Additional files

Additional file 1: Table S1. The location and SNP number for five susceptibility genes belonging to the network associated with leprosy. **Table S2.** The location and probe sets number for genes belonging to the subnetwork from *PI3K-AKT* signaling pathway and *Notch* signaling pathway. **Table S3.** Type I error rates of four methods (40 vertices and 54 edges in the network). **Table S4.** Type I error rates of four methods given the deviation from the normal distribution. **Figure S1.** A network including 40 vertices and 54 edges. **Figure S2.** The statistical power of the four methods under three scenarios. (a) only vertices change, (b) only edges change, (c) both vertices and edges change. (PDF 140 kb) Additional file 2:
**R code.** (DOCX 13 kb)

## Abbreviations

DC: Differentially connected
DE: Differentially expressed
GWAS: Genome-wide association study
KEGG: Kyoto encyclopedia of genes and genomes
*NetDifM*: Network difference measure
*NetDifM*pm: *NetDifM* based on permutation
*VEWDM*: Vertices and edges wise difference measure
