# Supplementary material for: A powerful score-based statistical test for group difference in weighted biological networks
Source: BMC Bioinformatics. 2016 Feb 12;17:86. doi: 10.1186/s12859-016-0916-x (PMC4751708; doi:10.1186/s12859-016-0916-x)
Supplement: Additional file 1: Table S1. — The location and SNP number for five susceptibility genes belonging to the network associated with leprosy. Table S2. The location and probe sets number for genes belonging to the subnetwork from PI3K-AKT signaling pathway and Notch signaling pathway. Table S3. Type I error rates of four methods (40 vertices and 54 edges in the network). Table S4. Type I error rates of four methods given the deviation from the normal distribution. Figure S1. A network including 40 vertices and 54 edges. Figure S2. The statistical power of the four methods under three scenarios. (a) only vertices change, (b) only edges change, (c) both vertices and edges change. (PDF 140 kb) [file 12859_2016_916_MOESM1_ESM.pdf]

Additional file 1

Table S1. The location and SNP number for 5 susceptibility genes belonging to the network associated with leprosy

Genes	Gene ID	SNP number	Location
CARD6	84674	197	5p13.1
HLA-DRB1	3123	246	6p21.3
RIPK2	8767	181	8q21
CARD9	64170	69	9q34.3
IFNG	3458	221	12q14

Table S2. The location and probe sets number for genes belonging to the subnetwork from *PI3K-AKT* signaling pathway and Notch signaling pathway

Genes	Gene ID	Probe sets number	Location
PI3K-AKT signaling pathway			
KRAS	3845	5	12P12.1
PIK3CA	5290	3	3q26.3
IRS1	3667	2	2q36
PDPK1	5170	4	16p13.3
AKT1	207	1	14q32.32
MDM2	4193	9	12q14.3-q15
TP53	7157	2	17p13.1
Notch signaling pathway			
DLL3	10683	4	19q13
DTX2	113878	1	7q11.23
CREBBP	1387	4	16p13.3
PTCRA	171558	3	6p21.3
JAG1	182	5	20p12.1-p11.23
DVL2	1856	2	17p13.1
SNW1	22938	3	14q24.3
HES1	3280	3	3q28-q29
RBPJ	3516	3	4p15.2
NOTCH2	4853	4	1p13-p11
PSENEN	55851	1	19q13.12
ADAM17	6868	3	2p25
NUMB	8650	4	14q24.3
NCOR2	9612	5	12q24

Table S3. Type I error rates of four methods (40 vertices and 54 edges in the network)

Sample size	*NetDifM*	*NetDifM*pm	*VEWDM*	*Yates'D*
200	0.014	0.057	0.050	0.048
400	0.043	0.052	0.045	0.058
600	0.044	0.055	0.051	0.059
800	0.040	0.053	0.054	0.049
1000	0.050	0.050	0.051	0.055

It is necessary to assess the performance of the proposed statistics, given the deviation from the normal distribution. For the network with 10 vertices and 21 edges, we designed the following two scenarios, (i) conduct the exponential transformation for 5 vertices randomly chosen among the 10 vertices; (ii) do the exponential transformation for all 10 vertices.

Table S4. Type I error rates of four methods given the deviation from the normal distribution.

Sample size	*NetDifM*	*NetDifM*pm	*VEWDM*	*Yates'D*
		scenario (i)		
100	0.011	0.041	0.048	0.054
200	0.024	0.042	0.046	0.039
300	0.036	0.051	0.047	0.048
500	0.042	0.056	0.054	0.046
800	0.042	0.053	0.046	0.050
		scenario (ii)		
100	0.009	0.051	0.058	0.048
200	0.018	0.045	0.055	0.047
300	0.028	0.050	0.048	0.054
500	0.027	0.046	0.054	0.058
800	0.040	0.055	0.057	0.045

The derivation of *NetDifM* based on the classical score test.

Let $Y = (y_1, y_2, \cdots, y_n)$ be an iid sample from a probability density function $\pi(x, \beta)$

For a generalized linear model $\log it[p(Y=1)] = \beta_0 + \beta_1 X$

The log-likelihood function is $L(\beta \mid x) = \sum_{l=1}^{N} \left(y_l \ln[\pi(x_l)] + (1 - y_l) \ln[1 - \pi(x_l)] \right)$

Hence, the score function is $U(\beta)$ where

$$U(\beta) = \frac{\partial L(\beta \mid x)}{\partial \beta} = \sum_{l=1}^{N} \left(y_i - \frac{\exp(\beta_0 + \beta_1 x_l)}{1 + \exp(\beta_0 + \beta_1 x_l)} \right) x_l$$

Under $H_0 : \beta_1 = 0$, the score statistics is

$$U(\hat{\beta}) = \sum_{l=1}^{N} \left(y_l - E(y) \right) x_l = \sum_{l=1}^{N} \left(y_l - \overline{Y} \right) x_l$$

Which can test the significance of the contribution of vertex x_i to the disease.

For the edge linked x_i and x_j, there contribution can be similarly obtained as

$$U(\hat{\beta}) = \sum_{l=1}^{N} (Y_l - \overline{Y})(x_{li} - \overline{x}_i)(x_{lj} - \overline{x}_j)$$

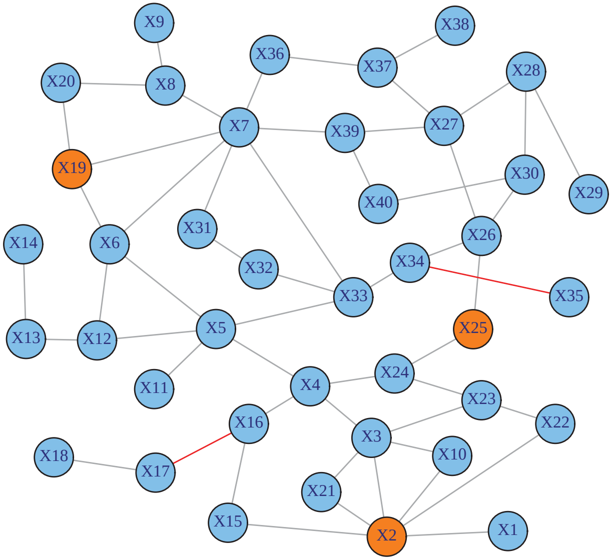

Figure S1 A network including 40 vertices and 54 edges.

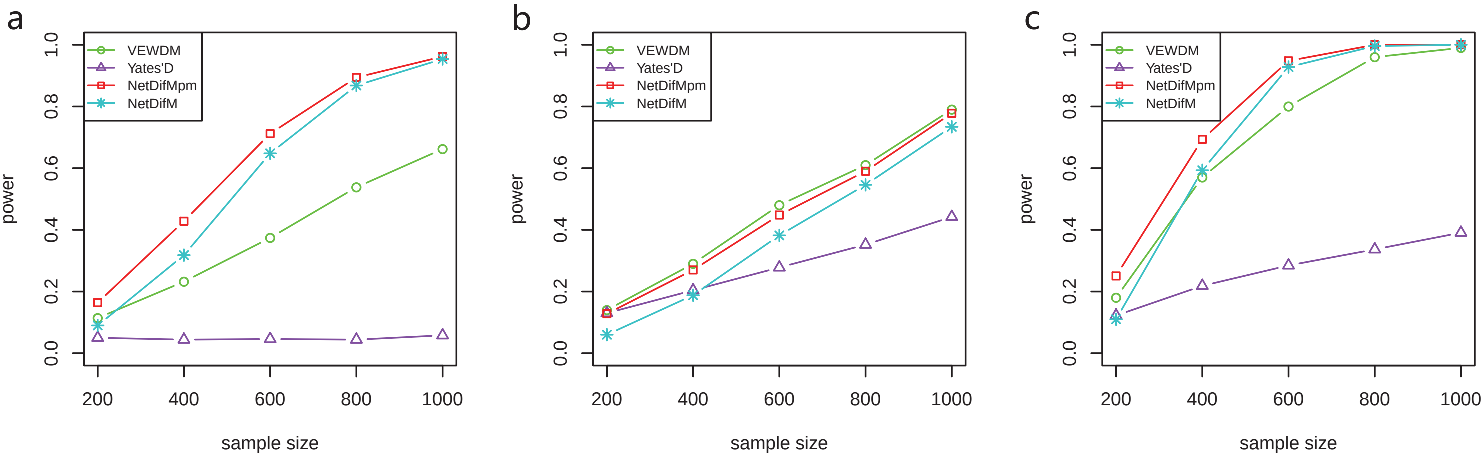

Figure S2. The statistical power of the four methods under three scenarios.(a) only vertices change, (b) only edges change, (c) both vertices and edges change
