# Supplementary material for: A powerful score-based statistical test for group difference in weighted biological networks
Source: BMC Bioinformatics. 2016 Feb 12;17:86. doi: 10.1186/s12859-016-0916-x (PMC4751708; doi:10.1186/s12859-016-0916-x)
Supplement: Additional file 2: — R code. (DOCX 13 kb) [file 12859_2016_916_MOESM2_ESM.docx]

**Additional file 2**

## netdif is an R function used to do test the group difference in weighted biological networks.

## The inputs are

# #group1 data matrix in group 1, columns of group1 are variables

## group2 data matrix in group 2

## adjmtrx adjacent matrix, nonzero value in adjmtrx indicating the corresponding edge will

# be detected

## method Choose one of the methods c("NetDifM","NetDifMpm","VEWDM"). By default

# the method is "NetDifM".

## permu Number of times to be used in permutation. By default the number is 1000.

# Ignored for "NetDifM".

## The output is the p-value

###############################################################

netdif <- function(adjmtrx, group1, group2, method="NetDifM", permu=1000) {

switch(method,

NetDifM = {

Pval <- netdifm(adjmtrx=adjmtrx,group1=group1,group2=group2)

},

NetDifMpm = {

Pval <- netdifpm(adjmtrx=adjmtrx,group1=group1,group2=group2,permu=permu)

},

VEWDM = {

Pval <- netVEWDM(adjmtrx=adjmtrx,group1=group1,group2=group2,permu=permu)

},

stop("Choose one of the methods \"NetDifM\",\"NetDifMpm\",\"VEWDM\".")

)

return(Pval)

}

###############################################################

netdifm <- function(adjmtrx,group1,group2) {

indw <- adjmtrx

case <- group1

contr <- group2

n1 <- nrow(group1)

n2 <- nrow(group2)

diag(indw) <- 0

ind <- indw != 0

adjmtrx[lower.tri(adjmtrx, diag = T)] <- 0

ind.rc <- which(!!adjmtrx,arr.ind=T)

cas.con <- rbind(case,contr)

cas.con.scal <- scale(cas.con,scale = F)

cas.con.cov <- cas.con.scal[ ,ind.rc[,1]] * cas.con.scal[ ,ind.rc[,2]]

cascontr <- cbind(cas.con,cas.con.cov)

sigma.cov <- cov(cascontr) * (1/n1 + 1/n2)

mean.d <- colMeans(cascontr[1:n1, ])

mean.c <- colMeans(cascontr[-c(1:n1), ])

mean.diff <- mean.d - mean.c

ujoint <- mean.diff %*% solve(sigma.cov) %*% mean.diff

df1 <- ncol(ind) + sum(ind)/2

NetDifM_p <- 1-pchisq(ujoint,df1)

c(NetDifM_p)

}

netdifpm <- function(adjmtrx,group1,group2,permu=permu) {

indw <- adjmtrx

case <- group1

contr <- group2

n1 <- nrow(group1)

n2 <- nrow(group2)

diag(indw) <- 0

ind <- indw != 0

adjmtrx[lower.tri(adjmtrx, diag = T)] <- 0

ind.rc <- which(!!adjmtrx,arr.ind=T)

cas.con <- rbind(case,contr)

ujoint.permu <- c(0)

cas.con.scal <- scale(cas.con,scale = F)

cas.con.cov <- cas.con.scal[ ,ind.rc[,1]] * cas.con.scal[ ,ind.rc[,2]]

cascontr <- cbind(cas.con,cas.con.cov)

sigma.cov <- cov(cascontr) * (1/n1 + 1/n2)

mean.d <- colMeans(cascontr[1:n1, ])

mean.c <- colMeans(cascontr[-c(1:n1), ])

mean.diff <- mean.d - mean.c

ujoint <- mean.diff %*% solve(sigma.cov) %*% mean.diff

for (j in 1:permu) {

id <- sample(1:(n1+n2),n1)

case <- cas.con[id,]

contr <- cas.con[-id,]

cas.con.pm <- rbind(case,contr)

cas.con.scal <- scale(cas.con.pm,scale = F)

cas.con.cov <- cas.con.scal[ ,ind.rc[,1]] * cas.con.scal[ ,ind.rc[,2]]

cascontr <- cbind(cas.con.pm,cas.con.cov)

mean.d <- colMeans(cascontr[1:n1, ])

mean.c <- colMeans(cascontr[-c(1:n1), ])

mean.diff <- mean.d - mean.c

ujoint.permu[j] <- mean.diff %*% solve(sigma.cov) %*% mean.diff

}

NetDifMpm_p <- mean(ujoint.permu > ujoint[1])

NetDifMpm_p

}

mt.test <- function(x,y,n1,n2) {

xmean <- colMeans(x)

ymean <- colMeans(y)

n12 <- n1+n2-2

sw <- sqrt(((n1-1)*diag(cov(x)) + (n2-1)*diag(cov(y)))/n12)

t <- (xmean - ymean)/(sw * sqrt(1/n1 + 1/n2))

p <- pt(t,n12)

pp <- rbind(p,1-p)

ptval <- 2*apply(pp,2,min)

rbind(t,ptval)

}

netVEWDM <- function(adjmtrx,group1,group2,permu=permu) {

indw <- adjmtrx

case <- group1

contr <- group2

n1 <- nrow(group1)

n2 <- nrow(group2)

nodes <- ncol(adjmtrx)

diag(indw) <- 0

ind <- indw != 0

r1 <- cor(case)

r2 <- cor(contr)

r1[!ind] <- 0

r2[!ind] <- 0

z0 <- (atanh(r1) - atanh(r2))/sqrt(1/(n1-3)+1/(n2-3))

mt <- mt.test(x=case,y=contr,n1=n1,n2=n2)

net.dif2 <- sum(mt[1,]^2)/nodes + sum(z0^2)/sum(ind)

cas.con <- rbind(case,contr)

net.dif2.permu <- c(0)

for (j in 1:permu) {

id <- sample(1:(n1+n2),n1)

case <- cas.con[id,]

contr <- cas.con[-id,]

r1 <- cor(case)

r2 <- cor(contr)

r1[!ind] <- 0

r2[!ind] <- 0

z.permu <- (atanh(r1) - atanh(r2))/sqrt(1/(n1-3)+1/(n2-3))

mt.pm <- mt.test(x=case,y=contr,n1=n1,n2=n2)

net.dif2.permu[j] <- sum(mt.pm[1,]^2)/nodes + sum(z.permu^2)/sum(ind)

}

pval <- mean(net.dif2.permu > net.dif2)

pval

}
